# Phenotypic and Functional Analysis of Human NK Cell Subpopulations According to the Expression of FcεRIγ and NKG2C

**DOI:** 10.3389/fimmu.2019.02865

**Published:** 2019-12-06

**Authors:** Kyung Hwan Kim, Hee Tae Yu, Ilwoong Hwang, Sungha Park, Su-Hyung Park, Sungjin Kim, Eui-Cheol Shin

**Affiliations:** ^1^Graduate School of Medical Science and Engineering, Korea Advanced Institute of Science and Technology, Daejeon, South Korea; ^2^Division of Cardiology, Department of Internal Medicine, Severance Cardiovascular Hospital, Yonsei University College of Medicine, Seoul, South Korea; ^3^Department of Microbiology and Molecular Genetics, Michigan State University, East Lansing, MI, United States; ^4^Department of Medical Microbiology and Immunology, Center for Comparative Medicine, University of California, Davis, Davis, CA, United States

**Keywords:** memory, NK cell, NKG2C, FcεRIγ, human, cytomegalovirus

## Abstract

Human memory-like NK cells are commonly defined by either a lack of FcεRIγ or gain of NKG2C expression. Here, we investigated the heterogeneity of human CD56^dim^ NK cell subpopulations according to the expression of FcεRIγ and NKG2C in a large cohort (*n* = 127). Although the frequency of FcεRIγ^−^ and NKG2C^+^ NK cells positively correlated, the FcεRIγ^−^ and NKG2C^+^ NK cell populations did not exactly overlap. The FcεRIγ^+^NKG2C^+^, FcεRIγ^−^NKG2C^+^, and FcεRIγ^−^NKG2C^−^ NK cell populations were only evident after HCMV infection, but each had distinct characteristics. Among the subpopulations, FcεRIγ^−^NKG2C^+^ NK cells exhibited the most restricted killer immunoglobulin-like receptor repertoire, suggesting clonal expansion. Moreover, FcεRIγ^−^NKG2C^+^ NK cells exhibited the lowest Ki-67 and highest Bcl-2 expression, indicating the long-lived quiescent memory-like property. Functionally, FcεRIγ^−^NKG2C^+^ NK cells had weak natural effector function against K562 but strong effector functions by CD16 engagement, whereas FcεRIγ^+^NKG2C^+^ NK cells had strong effector functions in both settings. Anatomically, the FcεRIγ^+^NKG2C^+^, FcεRIγ^−^NKG2C^+^, and FcεRIγ^−^NKG2C^−^ NK cell populations were present in multiple human peripheral organs. In conclusion, we demonstrate the heterogeneity of memory-like NK cells stratified by FcεRIγ and NKG2C and suggest both markers be utilized to better define these cells.

## Introduction

NK cells are cytotoxic innate lymphocytes responsible for early immune reactions to viral infections and tumors (1). Although immunological memory is a characteristic of adaptive immunity, emerging data indicate that NK cells can also acquire immunological memory (2, 3). Human NK cells can be classified in immature CD56^bright^ and mature CD56^dim^ cells (4, 5). Within the CD56^dim^ NK cell population, a subset of NK cells that gain NKG2C or lose FcεRIγ expression have been suggested as memory-like NK cells, which exhibit features of long-term persistence and unique epigenetic profiles (6, 7).

These memory-like NK cells are found exclusively in people infected with human cytomegalovirus (HCMV), and UL40 peptides have been described as specific antigens for the expansion of memory-like NK cells (6, 8). These memory-like NK cells can constitute a large proportion of the total NK cell population and persist for several years (6, 9). The role of these cells in human physiology is yet to be identified, but they are suggested to serve as effectors for controlling HCMV (10).

Memory-like NK cells have been studied more extensively in mouse models than human subjects. Although mouse and human memory-like NK cells share some characteristics, they also have distinct properties, including an absence of FcεRIγ^−^ cells in the mouse memory-like NK cell population (2, 6). Therefore, human-specific studies are required to better understand the biology of memory-like NK cells in humans. Although the expression of NKG2C and loss of FcεRIγ have been suggested to be key features of memory-like NK cells in humans (6, 7), NKG2C^+^ and FcεRIγ^−^ cells do not overlap exactly and are occasionally dissociated, implying heterogeneity within memory-like NK cells (11).

In the present study, we recruited a large cohort of adult donors to investigate the heterogeneity of human memory-like NK cells according to FcεRIγ and NKG2C expression. FcεRIγ^+^NKG2C^+^, FcεRIγ^−^NKG2C^+^, and FcεRIγ^−^NKG2C^−^ NK cells were only evident in HCMV-seropositive donors. FcεRIγ^+^NKG2C^+^, FcεRIγ^−^NKG2C^+^, and FcεRIγ^−^NKG2C^−^ NK cells exhibited distinct characteristics, both phenotypically and functionally. The FcεRIγ^−^NKG2C^+^ NK cell population had the most restricted killer cell immunoglobulin-like receptor (KIR) repertoire of all other subpopulations. Moreover, these cells exhibited characteristics of long-lived quiescent memory-like cells. Although FcεRIγ^−^NKG2C^+^ and FcεRIγ^−^NKG2C^−^ NK cells exhibited weak natural effector functions, FcεRIγ^+^NKG2C^+^ NK cells showed strong natural effector functions. However, FcεRIγ^−^NKG2C^+^ NK cells exerted strong effector functions by CD16 engagement. The memory-like NK cell subpopulations were detected in multiple human peripheral organs, but were less frequent in secondary lymphoid organs. These findings demonstrate the heterogeneity within memory-like NK cells and suggest that combining both markers may better define memory-like NK cells.

## Materials and Methods

### Human Subjects and Sample Collection

Human peripheral blood samples were collected from 127 Koreans who were recruited from subjects initially registered in the Yonsei Cardiovascular Genome cohort. The median age was 62 years (range, 20–81 years) and 81 were males. This study received prior approval from the Institutional Review Board of the Yonsei University College of Medicine (IRB number: 4-2001-0039, 4-2010-0500). All subjects gave written informed consent in accordance with the Declaration of Helsinki. Among the cohort, 123 subjects were seropositive for HCMV. Serial peripheral blood was obtained from an adult healthy donor with acute HCMV infection. Pre-infection peripheral blood mononuclear cells (PBMCs) were also available from this donor. Liver perfusates were obtained from healthy donor livers during liver transplantation, liver tissues from hepatitis B virus-infected explanted livers during liver transplantation, pleural fluid from patients with tuberculosis, tonsils were obtained during tonsillectomy, and lymph nodes without tumor involvement and tumor tissues were obtained during surgery for non-small cell lung cancer. PBMCs and liver sinusoidal lymphocytes (LSLs) were isolated from peripheral blood and liver perfusates, respectively, using standard Ficoll-Paque (GE Healthcare, Uppsala, Sweden) density gradient centrifugation. Tissues were dissociated into single cells using a gentleMACS dissociator (Miltenyi, Bergisch Gladbach, Germany) as described previously (12). The serological status for CMV, HSV1, HSV2, and EBV was measured using virus-specific ELISA kits (IBL International, Hamburg, Germany). All participants provided informed consent before enrollment.

### Flow Cytometry

Antibodies to the following surface molecules were used for cell staining: CD3 (HIT3a), CD56 (NCAM16.2), CD158b (CH-L), CD14 (MϕP9), CD19 (HIB19) (all from BD Biosciences, San Jose, CA), NKG2C (134591), CD158a (143211) (all from R&D Systems, Abingdon, UK), CD158e1 (DX9), Bcl-2 (100), Ki-67 (Ki-67) (all from BioLegend, San Diego, CA), CD57 (TBO1, eBioscience, San Diego, CA), and FcεRIγ (Merck Millipore, Billerica, MA). Dead cells were excluded using the LIVE/DEAD Fixable Red Dead Cell Stain Kit (Invitrogen, Carlsbad, CA). Intracellular staining for Ki-67, Bcl-2, and FcεRIγ was performed using a FoxP3 transcription factor staining buffer set (eBioscience, San Diego, CA) and specific antibodies. All samples were acquired on an LSR II cytometer and analyzed using FlowJo software version 10.4.0 (Treestar, San Carlos, CA).

### Functional Assays

PBMCs were thawed and rested overnight in RPMI supplemented with 10% fetal bovine serum and 1% penicillin/streptomycin. The PBMCs were co-cultured with K562 cells or anti-CD16-coated P815 cells for 12 h at an effector to target ratio of 10:1 in the presence of anti-CD107a (H4A3, BD Biosciences, San Jose, CA). Brefeldin A and monensin were added 1 h after co-incubation. For the anti-CD16 coating, P815 cells were incubated at 37°C with 10 μg/mL anti-CD16 antibody for 30 min. Cytokine production was detected by intracellular staining using antibodies to IFN-γ (B27) and TNF-α (Mab11) (all from BD Biosciences, San Jose, CA).

### Statistical Analysis

Statistical comparisons were performed as indicated in the figure legends. To quantify the diversity of KIRs, the inverse Simpson index was calculated, in which a lower value indicates less diversity. Two-sided *P* < 0.05 were considered significant. All statistical analyses were performed in Prism software version 6.0 (GraphPad, La Jolla, CA).

## Results

### Expression of FcεRIγ and NKG2C in Peripheral Blood CD56^dim^ NK Cells

**Figure 1:**
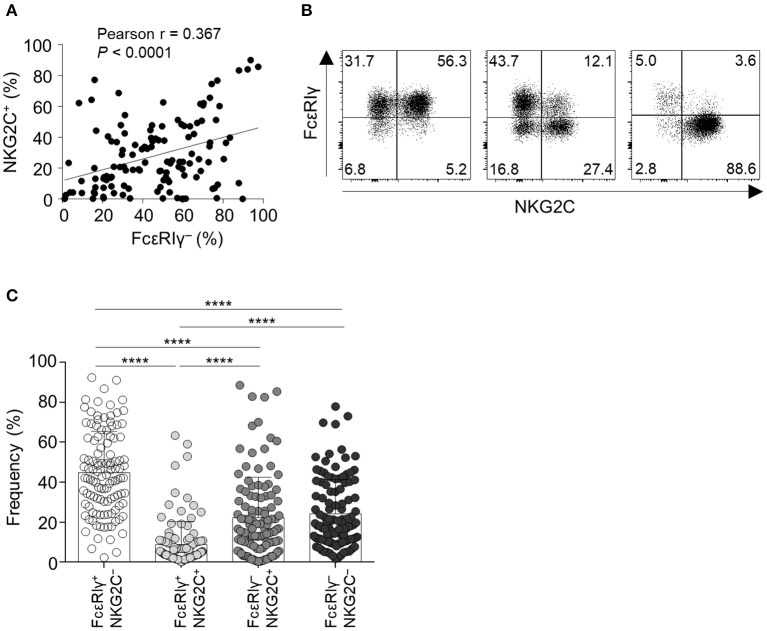
Expression of FcεRIγ and NKG2C among CD56^dim^ NK cells in peripheral blood. **(A)** Correlation of the frequency of FcεRIγ^−^ NK cells and NKG2C^+^ NK cells (*n* = 123). **(B)** Representative flow cytometry plots of FcεRIγ and NKG2C expression in gated CD56^dim^ NK cells from three HCMV-seropositive donors. **(C)** Frequency of the four subpopulations according to expression of FcεRIγ and NKG2C among CD56^dim^ NK cells in HCMV-seropositive donors (*n* = 123). Bar graphs indicate mean and s.d. Statistical analysis was performed by one-way ANOVA and *post-hoc* analysis by Tukey's multiple comparisons test **(C)**. Only significant differences are indicated. ****P < 0.0001.

First, we examined the expression of FcεRIγ and NKG2C in live CD56^dim^CD3^−^CD14^−^CD19^−^ cells (CD56^dim^ NK cells) among PBMCs from 123 HCMV-seropositive donors. The percentage of FcεRIγ^−^ NK cells significantly correlated with the percentage of NKG2C^+^ NK cells (Figure 1A). However, FcεRIγ^−^ cells were not always NKG2C^+^ and vice versa (Figure 1B). Among CD56^dim^ NK cells, the FcεRIγ^+^NKG2C^−^ population was most frequent and FcεRIγ^+^NKG2C^+^ population least frequent, whereas the FcεRIγ^−^NKG2C^+^ and FcεRIγ^−^NKG2C^−^ populations had similar frequencies (Figure 1C). In summary, the FcεRIγ^−^ and NKG2C^+^ populations overlap to some degree but are dissociated.

### FcεRIγ^−^NKG2C^+^ NK Cells Are Clonally Expanded From FcεRIγ^+^NKG2C^−^ NK Cells

**Figure 2:**
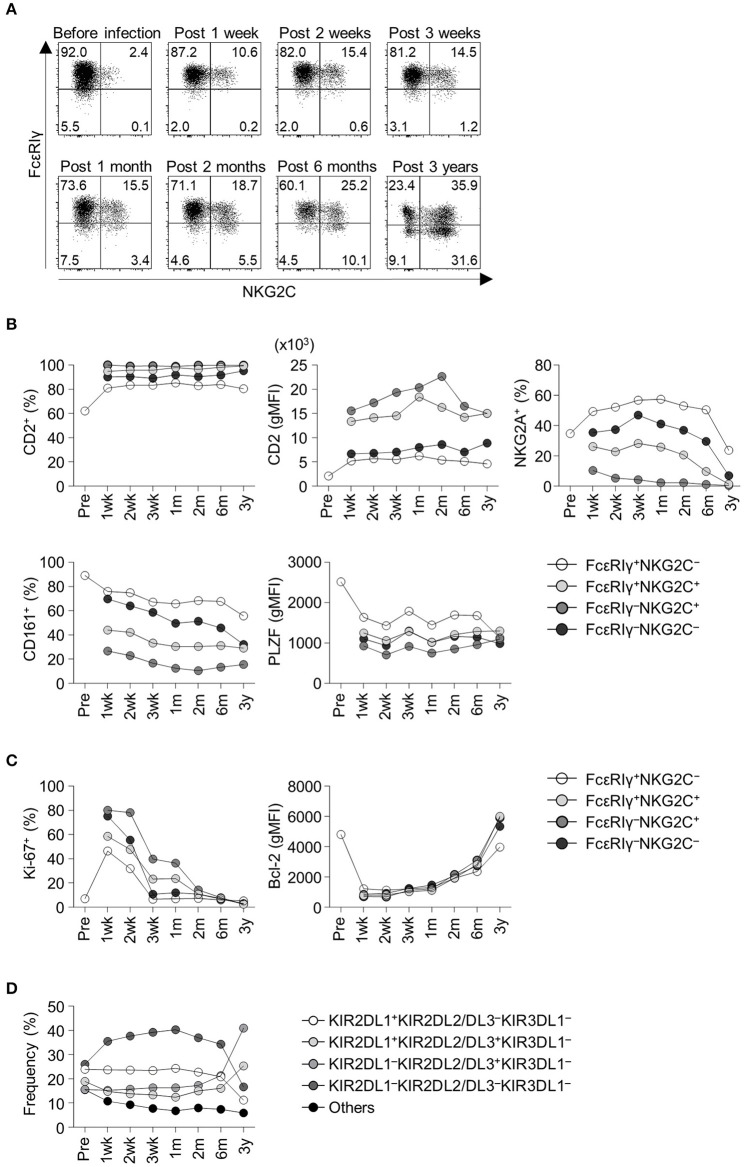
Frequency and phenotypes of CD56^dim^ NK cell subpopulations before and following acute HCMV infection. **(A)** Sequential change in FcεRIγ and NKG2C expression among CD56^dim^ NK cells in an adult healthy donor before and after acute HCMV infection. Time points indicate time from symptom onset. **(B)** Frequency of CD2^+^ cells, geometric mean fluorescence intensity (gMFI) of CD2, frequency of NKG2A^+^ cells, CD161^+^ cells, and gMFI of PLZF in FcεRIγ^+^NKG2C^−^, FcεRIγ^+^NKG2C^+^, FcεRIγ^−^NKG2C^+^, and FcεRIγ^−^NKG2C^−^ cells before and following acute HCMV infection. **(C)** Frequency of Ki-67^+^ cells and gMFI of Bcl-2 in FcεRIγ^+^NKG2C^−^, FcεRIγ^+^NKG2C^+^, FcεRIγ^−^NKG2C^+^, and FcεRIγ^−^NKG2C^−^ cells. **(D)** Frequency of KIR combinations before and following acute HCMV infection among CD56^dim^ NK cells.

We obtained serial peripheral blood from a healthy adult donor who experienced acute HCMV infection. Pre-infection PBMCs from this donor were also available from storage. Before HCMV infection, the donor had a low frequency of FcεRIγ^−^ NK cells and NKG2C^+^ NK cells (Figure 2A). Following acute HCMV infection, the FcεRIγ^+^NKG2C^+^ population appeared first, followed by the FcεRIγ^−^NKG2C^+^ population (Figure 2A). The frequency of FcεRIγ^+^NKG2C^+^ and FcεRIγ^−^NKG2C^+^ cells continuously increased for 3 years post-infection. The frequency of FcεRIγ^−^NKG2C^−^ cells also slightly increased. This representative example indicates that FcεRIγ^+^NKG2C^−^ cells first acquire NKG2C expression, and then subsequently lose FcεRIγ expression following acute HCMV infection.

Next, we analyzed relevant markers for memory-like NK cells during the course of acute HCMV infection in the healthy donor. Memory-like NK cells have been reported to have higher expression of CD2 (13) and lower expression of NKG2A, CD161, and PLZF (6, 7, 14). FcεRIγ^−^NKG2C^+^ cells exhibited high CD2 expression, low NKG2A^+^ and CD161^+^ cell frequency, and low PLZF expression early after acute HCMV infection (Figure 2B). During acute HCMV infection, all NK cell subpopulations showed a robust increase in the frequency of proliferating Ki-67^+^ cells and downregulation of Bcl-2 (Figure 2C). Among the subpopulations, FcεRIγ^−^NKG2C^+^, FcεRIγ^+^NKG2C^+^, and FcεRIγ^−^NKG2C^−^ cells, showed higher frequencies of Ki-67^+^ cells and higher expression of Bcl-2 than FcεRIγ^+^NKG2C^−^ cells (Figure 2C). We also analyzed the change in KIR repertoire during acute HCMV infection. KIRs are expressed on the surface of NK cells through a stochastic process, and the expression is maintained through DNA methylation (15, 16). This creates a diverse repertoire of NK cell clones characterized by individual combinations of KIRs. We investigated the expression of three commonly expressed KIRs, KIR2DL1, KIR2DL2/L3, and KIR3DL1, which resulted in eight KIR combinations. The KIR repertoire was skewed toward KIR2DL1^−^KIR2DL2/DL3^+^KIR3DL1^−^ within the CD56^dim^ NK cells, which was the dominant combination in the FcεRIγ^−^NKG2C^+^ subpopulation, following HCMV infection (Figure 2D).

**Figure 3:**
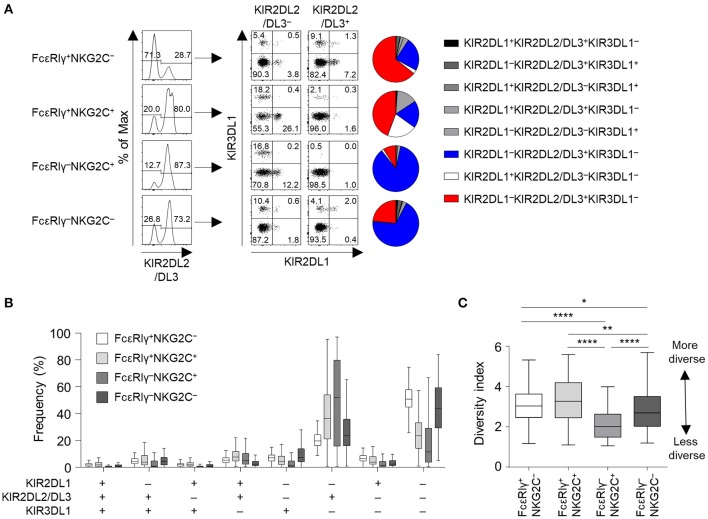
KIR repertoire of CD56^dim^ NK cell subpopulations. **(A)** Representative flow cytometry plots of KIR2DL1, KIR2DL2/DL3, and KIR3DL1 expression in FcεRIγ^+^NKG2C^−^, FcεRIγ^+^NKG2C^+^, FcεRIγ^−^NKG2C^+^, and FcεRIγ^−^NKG2C^−^ cells. The pie chart presents the distribution of the eight KIR subtypes. **(B)** Distribution of KIR subtypes in the FcεRIγ^+^NKG2C^−^, FcεRIγ^+^NKG2C^+^, FcεRIγ^−^NKG2C^+^, and FcεRIγ^−^NKG2C^−^ subpopulations (*n* = 123). **(C)** Diversity index of the KIR repertoire in the FcεRIγ^+^NKG2C^−^, FcεRIγ^+^NKG2C^+^, FcεRIγ^−^NKG2C^+^, and FcεRIγ^−^NKG2C^−^ subpopulations. The inverse Simpson index was calculated to evaluate diversity. The lines in the boxplot indicate median values, the boxes indicate IQR values, and the whiskers extend to 1.5× IQR values. Statistical analysis was performed by one-way ANOVA and *post-hoc* analysis by Tukey's multiple comparisons test **(C)**. Only significant differences are indicated. *P < 0.05; **P < 0.01; ****P < 0.0001.

We then analyzed the KIR repertoire in the four subpopulations of CD56^dim^ NK cells of the cohort of 123 HCMV-seropositive subjects. In a representative HCMV-seropositive donor, the KIR repertoire was most restricted in the FcRγ^−^NKG2C^+^ subpopulation, and the KIR2DL1^−^KIR2DL2/DL3^+^KIR3DL1^−^ combination predominated (Figure 3A). In the whole cohort, the four subpopulations had distinct patterns of KIR repertoire (Figure 3B). The KIR2DL1^−^KIR2DL2/DL3^−^KIR3DL1^−^ combination was more common in the FcεRIγ^+^NKG2C^−^ and FcεRIγ^−^NKG2C^−^ subpopulations, whereas the KIR2DL1^−^KIR2DL2/DL3^+^KIR3DL1^−^ combination was more common in the FcεRIγ^−^NKG2C^+^ and FcεRIγ^+^NKG2C^+^ subpopulations (Figure 3B). Furthermore, FcεRIγ^−^NKG2C^+^ cells exhibited the most restricted KIR diversity compared to the other subpopulations (Figure 3C). The FcεRIγ^−^NKG2C^−^ subpopulation also exhibited restricted KIR diversity compared to the FcεRIγ^+^NKG2C^−^ subpopulation. Taken together, the results indicate that FcεRIγ ^−^NKG2C^+^ NK cells may acquire their characteristics early after HCMV infection and may be clonally expanded from FcεRIγ^+^NKG2C^−^ NK cells.

### FcεRIγ^−^NKG2C^+^ NK Cells Exhibit Features of Long-Term Memory and Terminal Differentiation

**Figure 4:**
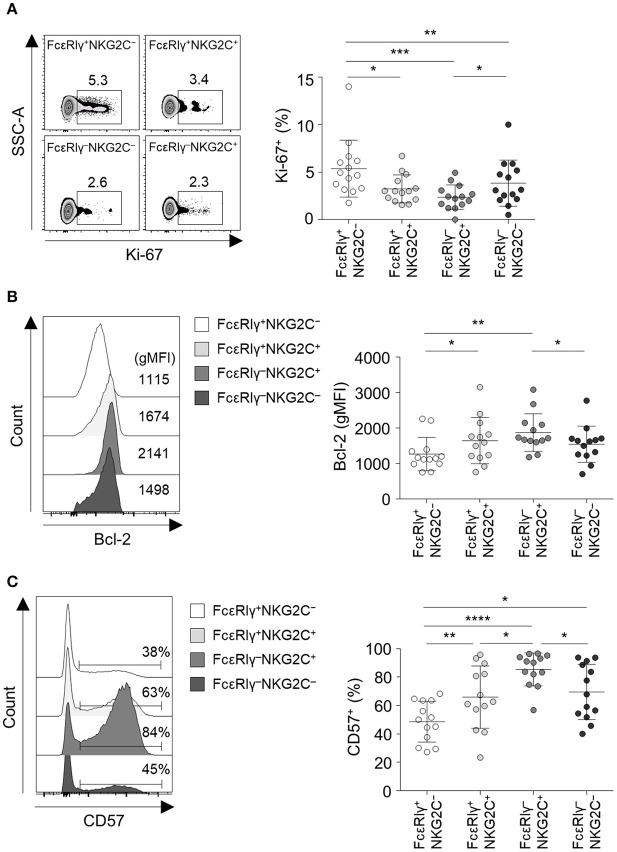
FcεRIγ^−^NKG2C^+^CD56^dim^ NK cells exhibit features of long-term memory and terminal differentiation. **(A)** Percentage of Ki-67^+^ cells among the FcεRIγ^+^NKG2C^−^, FcεRIγ^+^NKG2C^+^, FcεRIγ^−^NKG2C^+^, and FcεRIγ^−^NKG2C^−^ subpopulations (*n* = 13). **(B)** Geometric mean fluorescence intensity (gMFI) of Bcl-2 among the FcεRIγ^+^NKG2C^−^, FcεRIγ^+^NKG2C^+^, FcεRIγ^−^NKG2C^+^, and FcεRIγ^−^NKG2C^−^ subpopulations (*n* = 13). **(C)** Percentage of CD57^+^ cells among the FcεRIγ^+^NKG2C^−^, FcεRIγ^+^NKG2C^+^, FcεRIγ^−^NKG2C^+^, and FcεRIγ^−^NKG2C^−^ subpopulations (*n* = 13). Representative FACS plots are presented on the left **(A–C)**. Data are presented as mean ± s.d. Statistical analyses were performed by one-way ANOVA and *post-hoc* analysis by Tukey's multiple comparisons test **(A–C)**. Only significant differences are indicated. *P < 0.05; **P < 0.01; ***P < 0.001; ****P < 0.0001.

We further investigated the phenotype of the four different CD56^dim^ NK cell subpopulations. Long-lived memory CD8^+^ T cells are quiescent and apoptosis-resistant cells characterized by low Ki-67 and high Bcl-2 expression (17, 18). Similar to memory CD8^+^ T cells, FcεRIγ^−^NKG2C^+^ NK cells exhibited lower expression of Ki-67 (Figure 4A) and higher expression of Bcl-2 (Figure 4B) compared to the other subpopulations. FcεRIγ^+^NKG2C^+^ and FcεRIγ^−^NKG2C^−^ NK cells also exhibited lower expression of Ki-67 (Figure 4A), and FcεRIγ^+^NKG2C^+^ NK cells exhibited higher expression of Bcl-2 (Figure 4B) compared to FcεRIγ^+^NKG2C^−^ NK cells. Furthermore, FcεRIγ^−^NKG2C^+^ NK cells had the highest expression of CD57, which is a marker of highly mature and terminally differentiated NK cells (19), although FcεRIγ^+^NKG2C^+^ and FcεRIγ^−^NKG2C^−^ NK cells had upregulated CD57 expression compared to FcεRIγ^+^NKG2C^−^ NK cells (Figure 4C). These data indicate that FcεRIγ^−^NKG2C^+^ NK cells are more terminally differentiated than the other subpopulations and have features of long-lived memory cells.

### FcεRIγ^−^NKG2C^+^ NK Cells Have Reduced Activity Against K562 Target Cells but Enhanced CD16-Mediated Effector Functions

**Figure 5:**
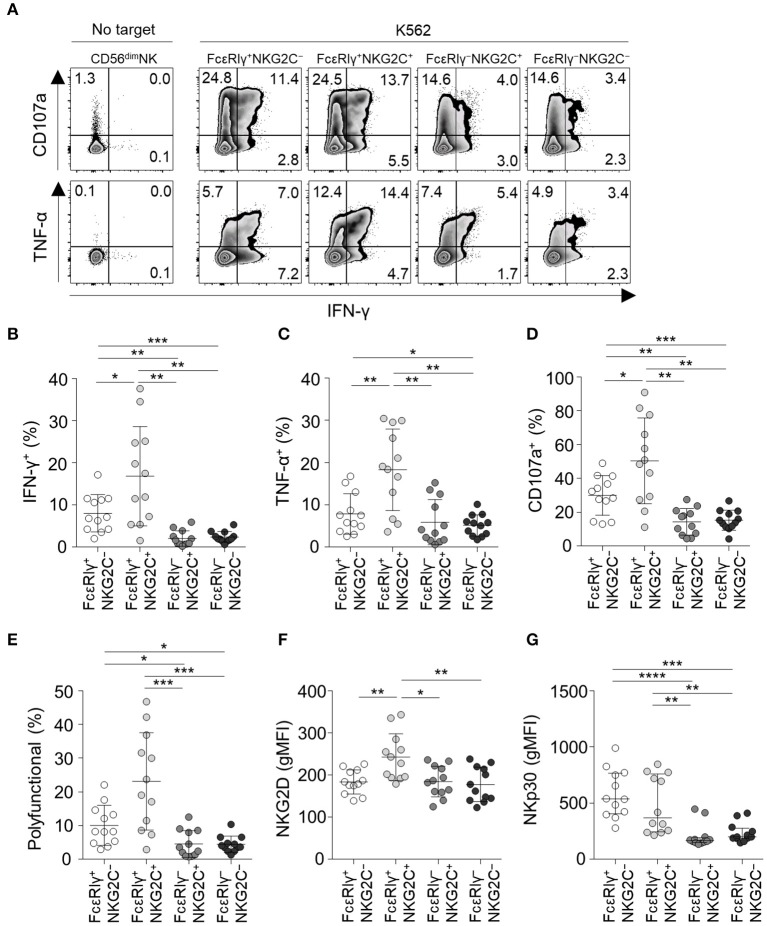
Effector functions of CD56^dim^ NK cell subpopulations in response to K562 target cells. **(A)** Representative flow cytometry plots of IFN-γ and TNF-α production, and CD107a expression after co-culture with K562 cells for 12 h among the FcεRIγ^+^NKG2C^−^, FcεRIγ^+^NKG2C^+^, FcεRIγ^−^NKG2C^+^, and FcεRIγ^−^NKG2C^−^ subpopulations. **(B–D)** Percentage of IFN-γ^+^ **(B)**, TNF-α^+^ **(C)**, and CD107a^+^ **(D)** cells among the FcεRIγ^+^NKG2C^−^, FcεRIγ^+^NKG2C^+^, FcεRIγ^−^NKG2C^+^, and FcεRIγ^−^NKG2C^−^ subpopulations (*n* = 12) after co-culture with K562 cells. **(E)** Percentage of polyfunctional cells that co-express IFN-γ, TNF-α, or CD107a. **(F,G)** gMFI of NKG2D **(F)** and NKp30 **(G)** among the FcεRIγ^+^NKG2C^−^, FcεRIγ^+^NKG2C^+^, FcεRIγ^−^NKG2C^+^, and FcεRIγ^−^NKG2C^−^ subpopulations. Statistical analyses were performed by one-way ANOVA and *post-hoc* analysis by Tukey's multiple comparisons test **(B–G)**. Only significant differences are indicated. *P < 0.05; **P < 0.01; ***P < 0.001; ****P < 0.0001.

To investigate the functionality of the four different subpopulations of CD56^dim^ NK cells, we measured cytokine production (IFN-γ and TNF-α) and degranulation (CD107a) in each subpopulation when co-cultured with K562 target cells (Figure 5A). FcεRIγ^−^NKG2C^+^ and FcεRIγ^−^NKG2C^−^ NK cells exhibited weak cytokine production and degranulation in response to K562 target cells (Figures 5A–D). In addition, FcεRIγ^−^NKG2C^+^ and FcεRIγ^−^NKG2C^−^ NK cells had minimal polyfunctionality, defined as cells simultaneously positive for IFN-γ, TNF-α, and/or CD107a (Figure 5E).

FcεRIγ^+^NKG2C^+^ NK cells had the highest functionality against K562 target cells. NKG2D receptor, which mediates the cytolytic activity of NK cells against target cells expressing NKG2D ligands (20), was most highly expressed in FcεRIγ^+^NKG2C^+^ NK cells, and expressed at significantly lower levels in FcεRIγ^−^NKG2C^+^ and FcεRIγ^−^NKG2C^−^ NK cells compared to FcεRIγ^+^NKG2C^+^ NK cells (Figure 5F). In addition, NKp30, which also has been documented in its function in killing B7-H6 expressing tumor cells (21), was more highly expressed in FcεRIγ^+^NKG2C^−^ and FcεRIγ^+^NKG2C^+^ NK cells (Figure 5G).

**Figure 6:**
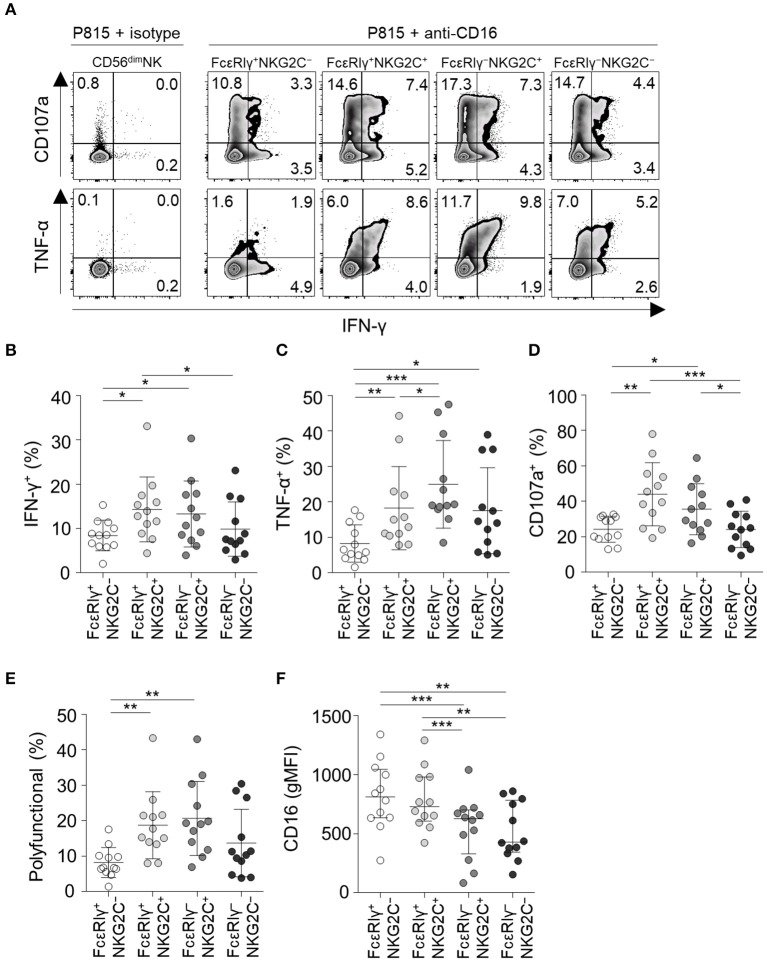
CD16-mediated effector functions of CD56^dim^ NK cell subpopulations. **(A)** Representative flow cytometry plots of IFN-γ and TNF-α production, and CD107a expression after co-culture with anti-CD16-coated P815 cells for 12 h among the FcεRIγ^+^NKG2C^−^, FcεRIγ^+^NKG2C^+^, FcεRIγ^−^NKG2C^+^, and FcεRIγ^−^NKG2C^−^ subpopulations. **(B–D)** Percentage of IFN-γ^+^ **(B)**, TNF-α^+^ **(C)**, and CD107a^+^ **(D)** cells among the FcεRIγ^+^NKG2C^−^, FcεRIγ^+^NKG2C^+^, FcεRIγ^−^NKG2C^+^, and FcεRIγ^−^NKG2C^−^ subpopulations (*n* = 12) after co-culture with anti-CD16-coated P815 cells. **(E)** Percentage of polyfunctional cells that co-express IFN-γ, TNF-α, or CD107a. **(F)** gMFI of CD16 among the FcεRIγ^+^NKG2C^−^, FcεRIγ^+^NKG2C^+^, FcεRIγ^−^NKG2C^+^, and FcεRIγ^−^NKG2C^−^ subpopulations. Statistical analyses were performed by one-way ANOVA and *post-hoc* analysis by Tukey's multiple comparisons test **(B–F)**. Only significant differences are indicated. *P < 0.05; **P < 0.01; ***P < 0.001.

Next, we analyzed NK cell functionality following co-culture with anti-CD16-coated P815 cells (Figure 6A). FcεRIγ^−^NKG2C^+^ NK cells had the highest TNF-α production among all subpopulations (Figure 6C) and higher IFN-γ production (Figure 6B) and degranulation activity (Figure 6D) than the FcεRIγ^+^NKG2C^−^ and FcεRIγ^−^NKG2C^−^ subpopulations. Moreover, FcεRIγ^−^NKG2C^+^ NK cells exhibited the highest polyfunctionality (Figure 6E). FcεRIγ^+^NKG2C^+^ NK cells also had higher cytokine production, degranulation, and polyfunctionality than FcεRIγ^+^NKG2C^−^ NK cells (Figures 6B–E). However, FcεRIγ^−^NKG2C^−^ NK cells exhibited higher TNF-α production than FcεRIγ^+^NKG2C^−^ NK cells (Figure 6C). Although the majority of CD56^dim^ NK cells express CD16, FcεRIγ^−^NKG2C^+^, and FcεRIγ^−^NKG2C^−^ subpopulations exhibited lower mean fluorescence intensity of CD16 (Figure 6F). Taken together, the results indicate that FcεRIγ^−^NKG2C^+^ NK cells have diminished K562-induced natural effector functions but enhanced CD16-mediated effector functions compared to other subpopulations.

### Virological, Demographic, and Anatomical Factors Associated With FcεRIγ^−^NKG2C^+^ NK Cells

**Figure 7:**
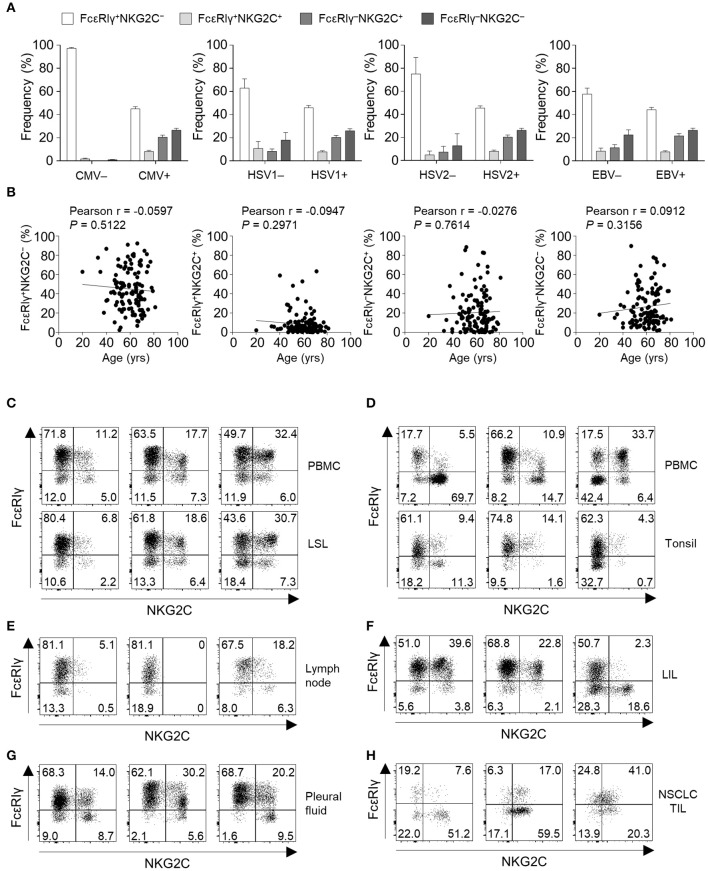
Virological, demographic, and anatomical factors associated with subpopulations of CD56^dim^ NK cells. **(A)** Frequency of FcεRIγ^+^NKG2C^−^, FcεRIγ^+^NKG2C^+^, FcεRIγ^−^NKG2C^+^, and FcεRIγ^−^NKG2C^−^ cells among CD56^dim^ NK cells according to serological status for HCMV (positive, *n* = 123; negative, *n* = 4), HSV1 (positive, *n* = 122; negative, *n* = 5), HSV2 (positive, *n* = 104; negative, *n* = 23), and EBV (positive, *n* = 122; negative, *n* = 5). The bars indicate mean and s.d. **(B)** Correlation between age and the frequency of FcεRIγ^+^NKG2C^−^, FcεRIγ^+^NKG2C^+^, FcεRIγ^−^NKG2C^+^, or FcεRIγ^−^NKG2C^−^ cells in HCMV seropositive donors (*n* = 123). **(C–H)** Representative FACS plots of FcεRIγ and NKG2C expression in gated CD56^dim^ NK cells among LSLs and paired PBMCs (*n* = 3; **C**), tonsils and paired PBMCs (*n* = 3; **D**), non-tumor involved lymph nodes (*n* = 3; **E**), liver-infiltrating lymphocytes (LILs; *n* = 3; **F**), pleural fluid (*n* = 3; **G**), and tumor-infiltrating lymphocytes (TILs) from patients with non-small cell lung cancer (NSCLC; *n* = 3; **H**).

To gain insights into the context in which the subpopulations of CD56^dim^ NK cells emerge, we investigated the correlation between the frequency of each subpopulations and virological and demographical factors in 127 donors. FcεRIγ^+^NKG2C^+^, FcεRIγ^−^NKG2C^+^, and FcεRIγ^−^NKG2C^−^ NK cells were not present in HCMV-seronegative individuals, whereas individuals seronegative for other herpesviruses had significant amounts of these populations. Among the 122 donors that were seropositive to HSV1, 121 were seropositive to HCMV and one donor that was seropositive to HSV1 but seronegative to HCMV had 2.68, 0.02, and 0.67% of FcεRIγ^+^NKG2C^+^, FcεRIγ^−^NKG2C^+^, and FcεRIγ^−^NKG2C^−^ cells among CD56^dim^ NK cells, respectively. All donors that were seropositive to HSV2 or EBV were seropositive to HCMV. These data suggest that HCMV drives the generation of FcεRIγ^+^NKG2C^+^, FcεRIγ^−^NKG2C^+^, and FcεRIγ^−^NKG2C^−^ NK cells (Figure 7A). Chronological aging has been shown to correlate with decreased naïve T-cell pools and increased memory T cells (22), but none of the CD56^dim^ NK cell subpopulations significantly correlated with age (Figure 7B).

Next, we examined the frequency of the four different CD56^dim^ NK subpopulations in peripheral organs (Supplementary Figure 1). FcεRIγ^+^NKG2C^+^, FcεRIγ^−^NKG2C^+^, and FcεRIγ^−^NKG2C^−^ NK cells were present at multiple sites, such as the liver sinusoid (Figure 7C), tonsils (Figure 7D), lymph nodes (Figure 7E), liver tissues (Figure 7F), pleural fluid (Figure 7G), and tumor tissues (Figure 7H). Matched PBMCs and LSLs were available from three donors, and the frequencies of FcεRIγ^+^NKG2C^+^, FcεRIγ^−^NKG2C^+^, and FcεRIγ^−^NKG2C^−^ NK cells were similar between the two compartments (Figure 7C). However, in tonsil tissues, the frequency of FcεRIγ^−^NKG2C^+^ NK cells was lower than in the matched PBMCs (Figure 7D). In addition, the frequency of FcεRIγ^−^NKG2C^+^ NK cells was relatively low in lymph nodes (Figure 7E). FcεRIγ^+^NKG2C^+^, FcεRIγ^−^NKG2C^+^, and FcεRIγ^−^NKG2C^−^ NK cells were also detected in liver tissues (Figure 7F), pleural fluid (Figure 7G), and tumor tissues (Figure 7H). These data suggest that FcεRIγ^+^NKG2C^+^, FcεRIγ^−^NKG2C^+^, and FcεRIγ^−^NKG2C^−^ NK cells are present in various human peripheral organs, and that FcεRIγ^−^NKG2C^+^ NK cells are preferentially present in non-lymphoid organs.

## Discussion

In the present study, we focused on the heterogeneity of human memory-like NK cells according to FcεRIγ and NKG2C expression and characterized these subpopulations. Although loss of FcεRIγ (6, 23, 24) and gain of NKG2C expression (25–27) have been suggested as markers of human memory-like NK cells in humans, we found that FcεRIγ^−^ NK cells and NKG2C^+^ NK cells do not exactly overlap and are rather dissociated, indicating the need for research based on both markers. Furthermore, we demonstrated that FcεRIγ^−^NKG2C^+^ NK cells exhibit typical features of long-lived quiescent memory-like cells with decreased function against K562 target cells but enhanced CD16-mediated effector capacity. The other subpopulations, FcεRIγ^+^NKG2C^+^ and FcεRIγ^−^NKG2C^−^ NK cells, exhibited intermediate characteristics between memory-like FcεRIγ^−^NKG2C^+^ and non-memory FcεRIγ^+^NKG2C^−^ NK cells.

The FcεRIγ^−^NKG2C^+^ NK cells had unique features compared to the other subpopulations. First, the FcεRIγ^−^NKG2C^+^ subpopulation was most clonally restricted in terms of the KIR repertoire. This is supported by a recent study demonstrating that NKG2C^+^ NK cells undergo clonal-like expansion by recognizing certain HCMV *UL40*-encoded peptides presented by HLA-E (25). Second, the FcεRIγ^−^NKG2C^+^ NK cells had the lowest Ki-67 expression and highest Bcl-2 expression, implying that this population has quiescent memory-like features. We also examined the expression of CD57, a marker of terminal differentiation (19, 28), and found that FcεRIγ^−^NKG2C^+^ cells most highly express CD57. The FcεRIγ^−^NKG2C^+^ NK cells were not only phenotypically unique, but also functionally unique. We found that the FcεRIγ^−^NKG2C^+^ subpopulation had strong CD16-mediated effector functions, especially in terms of TNF-α production and polyfunctionality. However, FcεRIγ^−^NKG2C^+^ NK cells exhibited weak cytokine production and degranulation activity against K562. These data from FcεRIγ^−^NKG2C^+^ NK cells coincide with previous results demonstrating that FcεRIγ^−^memory-like NK cells have an antibody-dependent, enhanced response to target cells (6, 7, 23, 24, 29). Overall, the phenotypical and functional analyses indicated that FcεRIγ^−^NKG2C^+^ NK cells are a proper memory-like NK cell population.

The FcεRIγ^+^NKG2C^+^ NK cell population exhibited different characteristics from the FcεRIγ^−^NKG2C^+^ NK cell population, though both populations expressed NKG2C. Despite the similarity in the KIR repertoires between both populations, FcεRIγ^−^NKG2C^+^ NK cells exhibited lower diversity than FcεRIγ^+^NKG2C^+^ NK cells, indicating further clonal expansion of FcεRIγ^−^NKG2C^+^ NK cells from FcεRIγ^+^NKG2C^+^ NK cells. In a donor with acute CMV infection, FcεRIγ^+^NKG2C^+^ NK cells first appeared after acute HCMV infection, followed by the appearance of FcεRIγ^−^NKG2C^+^ NK cells. FcεRIγ^−^NKG2C^+^ NK cells exhibited a robust increase in proliferation following HCMV infection and showed the most restricted KIR repertoire, supporting the clonal-like expansion of these cells. In addition, we found that characteristics of memory-like NK cells, such as high CD2 expression and low CD161, NKG2A, and PLZF expression (6, 7, 13, 14), were evident early after HCMV infection, suggesting that the epigenetic modification described in memory-like NK cells (6, 7) may take place during the early developmental period. Functionally, FcεRIγ^+^NKG2C^+^ NK cells exerted the highest effector function against K562 target cells among the NK subpopulations. Collectively, our data suggest that FcεRIγ^+^NKG2C^+^ NK cells may be effector cells against HCMV-infected cells that precede FcεRIγ^−^NKG2C^+^ NK cells.

Although the detailed mechanisms underlying the unique properties of memory-like NK cells have not been fully elucidated, FcεRIγ^−^or NKG2C^+^ NK cells were previously shown to have decreased expression of transcription factors (PLZF and Helios) and signaling molecules (SYK, EAT-2, and DAB-2) that are epigenetically regulated (6, 7). In the current study, we found a dissociation between the phenotypical and functional characteristics of NK subpopulations based on FcεRIγ or NKG2C expression. First, the weak K562-triggered effector function was more similar between the FcεRIγ^−^ NK cell populations (FcεRIγ^−^NKG2C^+^ and FcεRIγ^−^NKG2C^−^), whereas strong CD16-mediated effector functions were more similar between the NKG2C^+^ cell populations (FcεRIγ^+^NKG2C^+^ and FcεRIγ^−^NKG2C^+^). This implies that different mechanisms may be involved in the regulation of direct natural and antibody-dependent effector functions of memory-like NK cells. Notably, FcεRIγ^+^NKG2C^+^ NK cells, which had the highest activity against K562 cells, exhibited higher NKG2D and NKp30 expression, which are known to mediated K562 killing. The lower expression of NK receptors in FcεRIγ^−^NK cells was also documented in previous reports (7, 11). The expression of receptors responsible for each function may partially contribute to the distinct functionality of the NK subpopulations. However, FcεRIγ^−^NKG2C^+^ NK cells, which had the highest CD16-mediated effector functions, exhibited a relatively lower expression of CD16 compared to other subpopulations, which were also examined in a previous report (11). These findings suggest other mechanisms underlying its enhanced CD16-mediated effector functions. A possible explanation may be the lack of NK cell education, since lower fraction of the FcεRIγ^+^NKG2C^−^ and FcεRIγ^−^NKG2C^−^ were KIR^+^. In this context, it will also be helpful to examine the expression of NKG2A and monitor the functional responses in NKG2A^+^ vs. NKG2A^−^ cells expressing self or non-self KIRs.

The patterns of tissue localization of memory-like NK cells in humans have not been well-studied. However, in mouse models, the murine CMV (MCMV)-specific memory-like NK cells have been reported to be distributed in both lymphoid and non-lymphoid organs after MCMV infection (3). In human, CD49a^+^KIR^+^NKG2C^+^ NK cells have been described in liver tissue, but the expression of FcεRIγ was not examined (30). Although the number of evaluated donors was small, we found that, in humans, the frequency of memory-like NK cells, especially FcεRIγ^−^NKG2C^+^ NK cells, tends to be smaller in secondary lymphoid organs, such as lymph nodes and tonsils, compared to peripheral non-lymphoid organs. Notably, the frequency of FcεRIγ^−^NKG2C^−^ NK cells in the secondary lymphoid organs was relatively high compared to FcεRIγ^+^NKG2C^+^ and FcεRIγ^−^NKG2C^+^ NK cells, supporting the heterogeneity of the subpopulations in terms of organ distribution.

The specific role of memory-like NK cells in humans remains unclear. It is possible that memory-like NK cells are physiologically relevant in some contexts. A previous study showed that NKG2C^+^ NK cells expand in recipients of hematopoietic cell transplantation following HCMV reactivation (26). Moreover, memory-like NKG2C^+^ NK cells have been shown to be involved in the control of HCMV in kidney transplant recipients, implying the role of memory-like NK cells in controlling HCMV infection after organ transplantation (25). Recently, the presence of memory-like NK cells in patients co-infected with HCMV and HBV was reported (24). The antibody-dependent NK-cell response was enhanced in patients with HBV infection compared to healthy donors, though the clinical significance of this phenomenon needs to be researched further. The role of memory-like NK cells in the context of cancer has also been proposed. Expansion of memory-like NK cells after HCMV reactivation was associated with a reduced risk of leukemia relapse (31), and adoptively transferred memory-like NK cells demonstrated a robust clinical response in patients with myeloid leukemia (32). Moreover, a recent study showed that memory-like NK cells exhibit resistance to regulatory T-cell-mediated suppression, whereas the canonical NK cells were suppressed (8). Further investigation of the function and relevance of memory-like NK cells in various human diseases is required, including viral diseases and cancers.

This study had some limitations. We were not able to correlate HCMV viremia with the change in NK phenotypes during acute HCMV infection due to the lack of data on the virus titer. In addition, although paired PBMCs were available for liver sinusoidal lymphocytes and tonsil tissues, we did not have paired PBMCs for other peripheral tissues. The serology data of HCMV was not available for the donors of peripheral organs, although more than 97% of the Korean population is known to be seropositive to HCMV (33). Furthermore, we were not able to analyze the expression patterns of KIRs in accordance to the HLA-C genotype.

In conclusion, this study demonstrated heterogeneity within memory-like NK cells. The results indicate that both FcεRIγ and NKG2C should be utilized as markers to better define memory-like NK cells. This was also the first study to provide evidence of memory-like NK cells in diverse human peripheral organs, which will facilitate further research of memory-like NK cells in various contexts of human physiology and pathology.

## Data Availability Statement

All datasets generated for this study are included in the article/Supplementary Material.

## Ethics Statement

The studies involving human participants were reviewed and approved by Institutional Review Board of the Yonsei University College of Medicine. The patients/participants provided their written informed consent to participate in this study.

## Author Contributions

KK, HY, SK, and E-CS designed research. KK, HY, and IH performed research. KK, HY, SK, S-HP, and E-CS analyzed data. SP provided clinical samples. KK and E-CS wrote the paper.

### Conflict of Interest

The authors declare that the research was conducted in the absence of any commercial or financial relationships that could be construed as a potential conflict of interest.
