# Supplementary material for: Phenotypic and Functional Analysis of Human NK Cell Subpopulations According to the Expression of FcεRIγ and NKG2C
Source: Front Immunol. 2019 Dec 6;10:2865. doi: 10.3389/fimmu.2019.02865 (PMC6908468; doi:10.3389/fimmu.2019.02865)
Supplement: Supplementary file 1 [file Data_Sheet_1.PDF]

Supplementary Figure 1

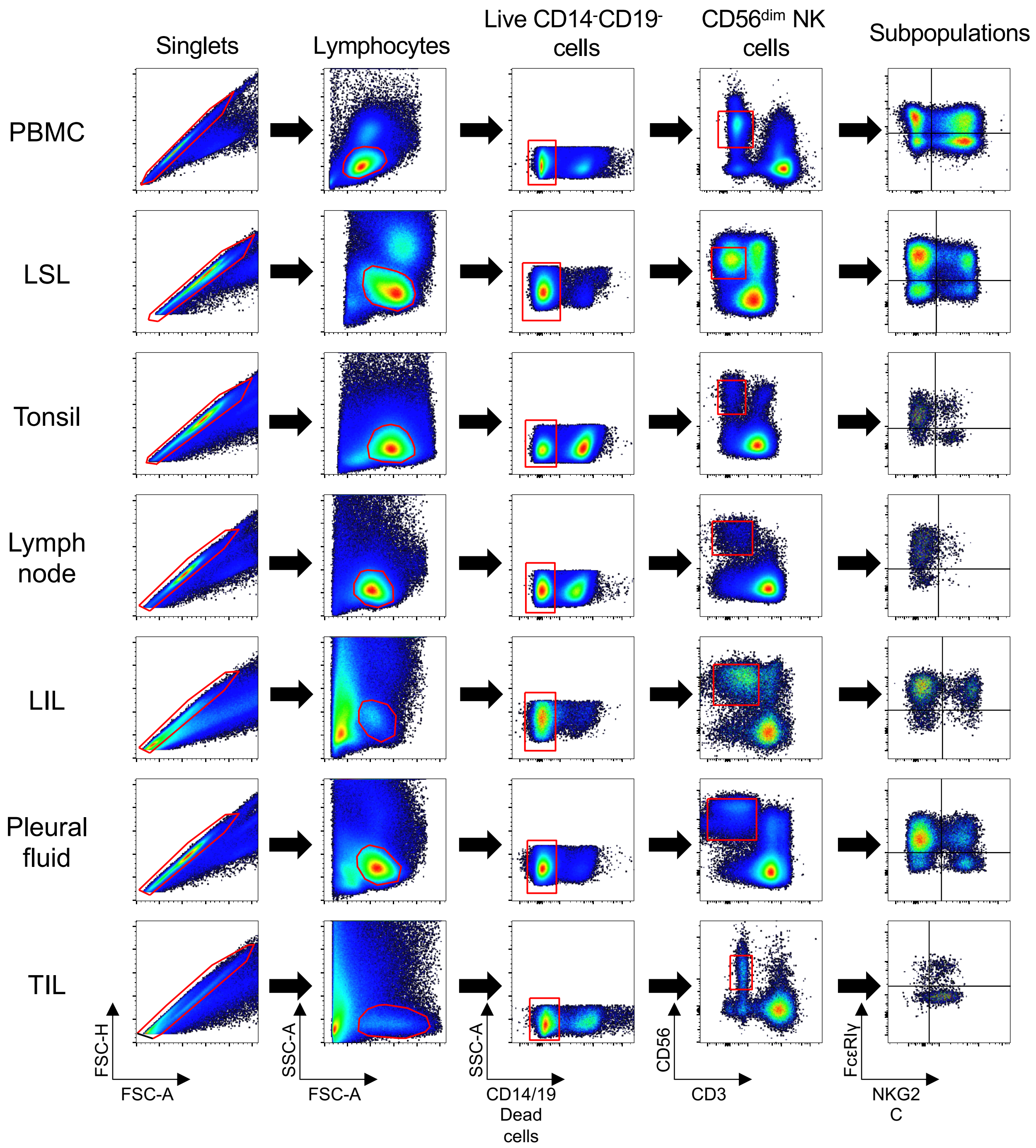

Supplementary Figure 1. Gating strategy of FcεRIγ⁺NKG2C⁻, FcεRIγ⁺NKG2C⁺, FcεRIγ⁻NKG2C⁺, or FcεRIγ⁻NKG2C⁻ CD56^{dim} NK cells in peripheral blood mononuclear cells (PBMCs), liver sinusoidal lymphocytes (LSLs), tonsils, lymph nodes, liver-infiltrating lymphocytes (LILs), pleural fluid, and tumor-infiltrating lymphocytes (TILs).
